# Radiologic assessment of third molar development and cervical vertebral maturation to validate age of majority in a Mexican population

**DOI:** 10.1007/s00414-025-03450-0

**Published:** 2025-02-19

**Authors:** Ana Belén Márquez-Ruiz, Lucas González-Herrera, Juan de Dios Luna, Aurora Valenzuela

**Affiliations:** 1 Department of Forensic Medicine, Faculty of Medicine, University of Granada, Avda. Doctor Jesús Candel Fábregas, 11, Granada, 18071 Spain; 2 Department of Statistics, Faculty of Medicine, University of Granada, Avda. Doctor Jesús Candel Fábregas, 11, Granada, 18071 Spain

**Keywords:** Forensic age estimation, Age of majority, Third molar, Cervical vertebrae, Computed tomography

## Abstract

An increase in irregular migration flows has further raised the importance of age estimation in forensic science. To improve the reliability of age of majority determination, one recommendation is to combine the evaluation of third molar development with age-related skeletal information. In the present study, we assessed mandibular third molar development and cervical vertebral maturation to evaluate the ability of these age indicators, alone and in combination, to accurately identify individuals 18 years of age or older. The study sample comprised 123 multi-slice computed tomography images of Mexican individuals (67 males and 56 females) aged between 14 and 22 years. Demirjian’s stages of tooth development and Baccetti’s stages of vertebral maturation were used. A fully developed mandibular third molar (stage H) indicated adult age with 100% certainty. However, around 70% of individuals older than 18 years had third molars that had not yet completed their development. Thus, immature third molars do not rule out the possibility that an individual has reached the age of majority. In the study sample, the combination of the maturity stages of teeth 38 and 48 or their combination with the cervical stages of maturation did not improve upon the prediction accuracy of either of the mandibular third molars alone (area under the ROC curve > 0.85). Therefore, these findings highlight the need to identify other complementary age estimation methods that minimize the number of false negatives (i.e., adults classified as minors) obtained with assessment of third molar development alone.

## Introduction

In recent decades, an increase in irregular migration flows has further raised the importance of age estimation in living juveniles and young adults. In many countries, the legal age limit is between 12 and 21 years, with 18 years of age the most frequent legal demarcation between minors and adults [1, 2].

Several methods have been recommended for forensic age diagnosis in criminal, civil, and asylum proceedings to determine whether an individual has reached the age of majority when the date of birth is uncertain. For example, the Study Group on Forensic Age Diagnostics (AGFAD) [3] recommends that age estimation be based on physical examination, radiographic examination of the left hand, dental development, and, when the ossification of the hand is complete, a radiographic study of the clavicles.

Regarding dental examination, the mineralization of third molars is relevant because they are the only teeth still growing during this period of life. Among the numerous methods developed to evaluate third molar maturation, the classification system of Demirjian et al. [4] appears to be the most reliable in terms of both observer agreement and the correlation between estimated and real age [1]. However, the third molar shows the most variable timing of maturation of all teeth [5]. Thus, for a more reliable radiographic estimation of age, several authors have recommended a combined assessment of third molar development with age-related skeletal information [3, 5]. Nevertheless, when dental and skeletal parameters are combined for age estimation in living individuals, forensic professionals must consider ethical issues related to the biological effects of radiation exposure [6].

Accordingly, cervical vertebral development has been proposed for age estimation in combination with third molar development [5–8]. Cervical vertebral development has been studied by several authors, who have pointed out the morphological changes that occur in the different maturational stages [9, 10]. In addition, dental (third molar) and skeletal (cervical vertebrae) maturation can be evaluated in the same radiological study (e.g., lateral cephalogram) [11], without additional exposure to the patient.

Therefore, the aims of the present study were (1) to assess mandibular third molar development and cervical vertebral maturation in a population sample of individuals of known chronological age and sex, and (2) to evaluate the ability of these age indicators, alone and in combination, to accurately identify individuals 18 years of age or older. We also evaluated the usability of computed tomography scans for this purpose, because most previous studies used panoramic and cephalometric radiographs to assess third molar development and cervical vertebral maturation, respectively.

## Materials and methods

The sample comprised multi-slice computed tomography (MSCT) images of 123 individuals aged between 14 and 22 years (67 males: age range, 14.00–22.83 years; mean, 17.96 years; 56 females: age range, 14.00–22.92 years; mean, 18.06 years) acquired for clinical reasons and collected retrospectively from the database of the Diagnostic and Image Center of University Hospital Dr. Eleuterio González, Autonomous University of Nuevo León (AUNL), in Monterrey, Mexico. A multi-slice helical high-resolution LightSpeed^®^ VCT CT system (General Electric Healthcare, Chicago, IL, USA) was used to obtain the CT images, and files were saved in DICOM format. MSCT scans were excluded if both mandibular third molars were missing or if an image deformity affected the area of interest. Numbers of individuals by age group and sex are shown in Table 1.

**Table 1:** Age and sex distribution of the study sample (*n* = 123)

Age (years)	Males	Females	Total
14–14.99	8	8	16
15–15.99	10	10	20
16–16.99	8	6	14
17–17.99	11	5	16
18–18.99	7	5	12
19–19.99	8	5	13
20–20.99	5	7	12
21–21.99	3	4	7
22–22.92	7	6	13
Total	67	56	123

Each image was labeled with a random number to blind the observers to the patient’s name, sex, date of birth, and date of the MSCT image. The chronological age of each case was calculated from the date of birth and the date of the MSCT image. The protocols specifying the collection of human MSCT images were approved by the Ethic Committees on Human Research of AUNL University Hospital and the University of Granada (3342/CEIH/2023), and the study was conducted in accordance with the ethical standards laid down by the Declaration of Helsinki. All patients, their parents, or their legal representatives, as appropriate, had previously consented to the use of these clinical images for research purposes.

The mineralization stage of each mandibular third molar was evaluated according to the mineralization scheme proposed by Demirjian et al. [4] and adapted by Mincer et al. [12]. This system is based on eight stages of tooth formation ranging from the beginning of cusp calcification (stage A) to root apex closure (stage H). To assess the maturational status of the cervical vertebrae (C2, C3, and C4), the Cervical Vertebral Maturation (CVM) method of Baccetti et al. [9] was used. This method is based on six stages of maturation, ranging from stage 1, where the borders of C2, C3, and C4 are flat and the bodies of both C3 and C4 are trapezoidal, to stage 6, where the lower borders of C2, C3, and C4 are evident and at least one of the bodies of C3 and C4 is rectangular vertical in shape.

To test agreement between observers, mandibular third molars (teeth 38 and 48, according to FDI notation) and cervical vertebrae (C2, C3, and C4) were evaluated by two trained, independent observers: a forensic odontologist (A.B.M.R.) and an odontologist with clinical experience (L.L.) for third molars, and the same forensic odontologist (A.B.M.R.) and a physician (M.P.) for cervical vertebrae evaluation. Mineralization scores obtained by only one of the observers (the forensic odontologist), who had the highest level of experience and training, were used for the results reported here. To test intra-observer variability, all MSCT images were re-examined by this observer 4 weeks after the initial scoring to assess agreement.

Statistical analysis was conducted with Stata version 14.1 software (StataCorp LLC, College Station, TX, USA). Descriptive statistics were recorded and are expressed as the mean ± standard error of the mean. Intra- and inter-observer agreement for mineralization stages was determined as the quadratic weighted kappa coefficient [13]. Differences in mineralization scores between mandibular third molars of either side of the jaw were determined with a t-test. A value of *p* < 0.05 was considered statistically significant. Method accuracy was determined by receiver operating characteristic (ROC) analysis, which combines the concepts of sensitivity and specificity into a single measure of accuracy defined as the area under the ROC curve (AUC) [14]. The 95% confidence interval (CI) was calculated for each value.

## Results

Demirjian’s stages of tooth development and Baccetti’s stages of vertebral maturation were determined for mandibular third molars and cervical vertebrae (C2, C3, and C4), respectively. The kappa values for intra-observer agreement were 0.98 (95% CI: 0.79–1.00) for tooth 38, 0.98 (95% CI: 0.79–1.00) for tooth 48, and 0.89 (95% CI: 0.72–1.00) for cervical vertebrae. The kappa values for inter-observer agreement were high for both third molars: 0.90 (95% CI: 0.72–1.00) for tooth 38 and 0.91 (95% CI: 0.73–1.00) for tooth 48. For cervical vertebrae, the inter-observer agreement was 0.78 (95% CI: 0.60–0.95).

Table 2 presents the mean age for each Demirjian stage and by sex for both mandibular third molars. Stages below B for tooth 38 and C for tooth 48 were not observed. As can be seen, females reached stages D and E at younger ages than males. However, the mean ages were younger for males than for females at stages F and G, with a difference of around 2 years, while the mean ages were similar for both sexes at stage H. Analyses based on percentiles (Table 2) showed a faster rate of mineralization in males, except in stage D (final stage of crown formation). The results were uniform for both hemiarches because, regardless of sex, no significant differences were found in mineralization patterns between third molars from either side of the mandible (*p* > 0.05).

**Table 2:** Mean (± standard error), minimum and maximum age, and age distribution (expressed in percentiles) by Demirjian’s stage of tooth development and sex

Tooth	Stage, sex	*n*	Mean ± SE	Minimum–Maximum	Percentiles		
					50	75	90
38	B, males	1	14.75 ± 0.00	14.75–14.75	14.75	14.75	14.75
	C, males	5	14.35 ± 0.19	14.00–15.08	14.25	14.71	-
	C, females	5	15.82 ± 0.57	14.00–17.50	16.00	16.83	-
	D, males	5	15.92 ± 0.71	14.00–18.08	15.67	17.46	-
	D, females	11	15.33 ± 0.27	14.08–16.83	15.25	15.92	16.72
	E, males	10	17.28 ± 0.49	15.33–19.58	17.29	18.69	19.57
	E, females	11	16.98 ± 0.75	14.00–21.50	16.33	19.00	21.37
	F, males	10	17.04 ± 0.75	14.75–22.83	16.25	18.00	22.45
	F, females	6	18.94 ± 0.41	17.75–20.50	18.71	19.94	-
	G, males	21	18.16 ± 0.38	14.83–20.92	17.92	19.71	20.82
	G, females	14	20.51 ± 0.43	17.83–22.75	20.50	22.12	22.63
	H, males	11	21.47 ± 0.40	18.42–22.83	22.00	22.42	22.82
	H, females	4	21.65 ± 0.80	19.50–22.92	22.08	22.90	-
48	C, males	8	15.02 ± 0.45	14.00–17.17	14.50	16.40	-
	C, females	7	15.62 ± 0.41	14.00–17.50	15.42	16.17	-
	D, males	5	17.17 ± 0.86	14.75–19.50	17.83	18.79	-
	D, females	14	15.76 ± 0.27	14.08–17.42	15.83	16.46	17.38
	E, males	6	16.94 ± 0.52	15.83–19.17	16.67	17.85	-
	E, females	7	16.67 ± 1.02	14.00–20.83	15.67	19.75	-
	F, males	11	16.77 ± 0.42	15.33–19.00	16.50	18.42	18.97
	F, females	6	18.62 ± 0.82	14.83–20.50	18.92	20.13	-
	G, males	19	18.21 ± 0.36	14.83–20.92	17.92	19.58	20.75
	G, females	16	20.41 ± 0.40	17.83–22.75	20.50	21.81	22.58
	H, males	11	21.27 ± 0.39	18.42–22.83	21.50	22.08	22.82
	H, females	5	21.77 ± 0.63	19.50–22.92	22.25	22.88	-

*n* number of cases, *SE* standard error

Regarding CVM stages (Table 3), stages below 4 were not found in the sample population. Mean ages were younger for males than for females at CVM stages 4 and 6. At CVM stage 5, the mean age was around 9 months younger for females than for males. Similar results were shown for percentiles (Table 3), with earlier mineralization in males than in females, except for CVM stage 5. Notably, although mean ages and percentiles increased with the CVM stage in both sexes, each of the CVM stages almost entirely covered the complete age spectrum of the study population.

**Table 3:** Mean (± standard error), minimum and maximum age, and age distribution (expressed in percentiles) by CVM stage and sex

CVM stage, sex	*n*	Mean ± SE	Minimum– Maximum	Percentiles		
				50	75	90
4, males	14	15.57 ± 0.41	14.00–18.83	15.17	16.67	18.21
4, females	9	16.61 ± 0.97	14.08–22.25	15.08	19.00	-
5, males	24	18.21 ± 0.48	14.83–22.83	17.88	19.83	21.79
5, females	26	17.25 ± 0.49	14.00–22.50	16.29	19.19	21.63
6, males	29	18.92 ± 0.44	15.08–22.83	18.58	21.17	22.42
6, females	21	19.69 ± 0.50	14.00–22.92	19.83	21.38	22.82

*n* number of cases, *SE* standard error

Tables 4 and 5 present the number of individuals by age group and sex and the likelihood of an individual being 18 years of age or older based on the stage of maturation of the mandibular third molar (Table 4) or the cervical vertebrae (C2, C3, and C4) (Table 5). In the first age group (14–15.99 years), individuals classified in lower maturity stages comprised the largest proportion, and this percentage decreased as the degree of mineralization increased. In contrast, in the last age group (individuals ≥ 18 years of age), most individuals were in the most advanced stages of mineralization.

**Table 4:** Number of individuals by age group and the likelihood of an individual being at least 18 years of age according to Demirjian’s stage of tooth development and sex

Tooth	Stage, sex	Age group^a^			Probability of being age ≥ 18
		14.00–15.99	16.00–17.99	≥ 18	
38	B, males	1	0	0	0.000
	C, males	5	0	0	0.000
	C, females	2	3	0	0.000
	D, males	3	1	1	0.200
	D, females	9	2	0	0.000
	E, males	3	4	3	0.300
	E, females	5	3	3	0.273
	F, males	4	4	2	0.200
	F, females	0	1	5	0.833
	G, males	2	9	10	0.476
	G, females	0	1	13	0.929
	H, males	0	0	11	1.000
	H, females	0	0	4	1.000
48	C, males	6	2	0	0.000
	C, females	4	3	0	0.000
	D, males	2	1	2	0.400
	D, females	9	5	0	0.000
	E, males	2	3	1	0.007
	E, females	4	1	2	0.286
	F, males	4	4	3	0.273
	F, females	1	0	5	0.833
	G, males	1	9	9	0.474
	G, females	0	1	15	0.938
	H, males	0	0	11	1.000
	H, females	0	0	5	1.000

^a^Age expressed in years

**Table 5:** Number of individuals by age group and the likelihood of an individual being at least 18 years of age according to CVM stage and sex

CVM stage, sex	Age group^a^			Probability of being age ≥ 18
	14.00–15.99	16.00–17.99	≥ 18	
4, males	9	4	1	0.071
4, females	6	1	2	0.222
5, males	5	8	11	0.458
5, females	11	8	7	0.269
6, males	4	7	18	0.621
6, females	1	2	18	0.857

^a^Age expressed in years

We used ROC analysis to determine the accuracy of the examiner’s ability to correctly estimate age as younger or older than 18 years based on Demirjian’s stages of mineralization and/or CVM. To avoid reducing the sample size and because there were no significant differences in mineralization patterns between mandibular third molars from either side of the jaw, the mineralization stage of the contralateral tooth was considered if one of the molars was absent. Table 6 shows the criterion validity for an individual being at least 18 years of age in relation to mineralization scores, along with sensitivity and specificity values for different cut-off points and the AUCs for each age indicator.

**Table 6:** Sensitivity, specificity, and AUC values for each age indicator at different cut-off points

Age indicator	Cut-off point^a^	Sensitivity^b^	Specificity^b^	AUC^b^
Tooth 38	E/FGH	0.86 (0.74–0.94)	0.68 (0.56–0.79)	0.858 (0.797–0.918)
	EF/GH	0.70 (0.57–0.82)	0.82 (0.70–0.90)	
	EFG/H	0.28 (0.17–0.42)	1.00 (0.95–1.00)	
Tooth 48	E/FGH	0.90 (0.79–0.96)	0.67 (0.54–0.78)	0.875 (0.818–0.931)
	EF/GH	0.74 (0.60–0.85)	0.83 (0.72–0.91)	
	EFG/H	0.32 (0.20–0.45)	1.00 (0.95–1.00)	
Cervical vertebrae (C2, C3, and C4)	4/56	0.95 (0.85–0.99)	0.30 (0.20–0.43)	0.745 (0.665–0.825)
	45/6	0.63 (0.49–0.76)	0.79 (0.67–0.88)	

^a^Cut-off points are expressed as Demirjian’s mineralization stages (E, F, G, and H) and CVM stages (4, 5, and 6). The stages of development located to the right of the slash classify individuals as 18 years of age or older

^b^Results are expressed as the mean and 95% confidence interval

The combination of the maturity stages of teeth 38 and 48 did not improve upon the prediction accuracy of the third molars alone (AUC < 0.80), although the specificity was somewhat better for the cut-off point that classified an individual as being ≥ 18 years of age if both mandibular third molars were at stage G or H (EF/GH): 0.85 (95% CI: 0.74–0.93), with a sensitivity of 0.70 (95% CI: 0.51–0.82). The combination of mandibular third molars and cervical vertebrae also did not lead to a better estimate of the age of majority (AUC < 0.85). However, higher specificity values—0.89 (95% CI: 0.79–0.96) and 0.88 (95% CI: 0.78–0.95), respectively—were found for cut-off points that classified individuals as being ≥ 18 years of age if their mandibular third molars were at stage F, G, or H and their cervical vertebrae were at CVM stage 6 (E/FGH + 45/6) and that classified individuals as being ≥ 18 years if their mandibular third molars were at stage G or H and their cervical vertebrae were at CVM stage 5 or 6 (EF/GH + 4/56). The sensitivity values for these cut-off points were 0.53 (95% CI: 0.39–0.66) and 0.67 (95% CI: 0.53–0.79), respectively.

## Discussion

Determining whether a person has reached 18 years of age is a challenging task for forensic practitioners. Estimating the age of majority of living individuals is critical in cases of unaccompanied minors, illegal immigration, asylum seekers, or criminal liability because incorrect estimates can lead to ethical (minors classified as adults) and technical (adults classified as minors) errors [15]. Therefore, accurate and reliable methods are required.

The reliability of the methods employed in this study for assessing the stage of maturation of mandibular third molars and cervical vertebrae was verified by intra- and inter-observer agreement studies. The intra-observer agreement for the evaluation of the mineralization stages of mandibular third molars and cervical vertebrae was “almost perfect” according to the six-category system proposed by Landis and Koch [16]. Our inter-observer values for Demirjian’s stages of mineralization were also remarkably high (> 0.9). However, for Baccetti’s cervical stages, the inter-observer agreement was just “substantial” [16]. These results are similar to those obtained by others, with Demirjian’s method for the stage assessment of third molars showing very good intra- and inter-observer agreements [17–19] and Baccetti’s method displaying lower reproducibility [8, 20].

Although we calculated the mean age for each stage of mandibular third molar development (Table 2) and CVM (Table 3), comparisons with previous results were difficult because the minimum and maximum ages of enrolled participants vary between studies. In this regard, it is important to consider the upper age limit, because individuals may be classified in the final stage of development at any age after the complete mineralization [21]. Mean ages are therefore influenced by the maximum age and are not always ideal indicators of early or late maturation [22]. However, they were useful for the assessment of differences between the sexes in the study sample.

Our findings (Table 2) were consistent with those of earlier studies that reported differences between the sexes in the development of mandibular third molars, with root formation occurring earlier in males than in females [17, 23, 24]. In addition, there were no significant differences in mandibular third molar development between the left and right sides, as reported in most previous studies [18, 19, 22–25]. However, the mandibular third molars of an individual can be at different stages of development, resulting in a Demirjian’s stage different from that of the contralateral tooth. This might explain why, in our male sample, the mean age at stage D for tooth 48 was older than the mean age at the same stage for tooth 38 and was even older than the mean ages at stages E and F for tooth 48. Therefore, these findings show the variability of the third molar with respect to its time of formation.

Regarding cervical vertebral maturation, early formation was found in males in prior studies [8], while others reported that skeletal maturation occurs earlier in females than in males [7, 26, 27]. In our sample, while males matured completely earlier than females, females reached the final stage of potential cervical vertebral development (CVM stage 5) at younger ages (Table 3).

Although socioeconomic factors are often indicated as the most important variable in dental and skeletal maturation differences [10, 25], sexual differences, in addition to ethnic/geographical variations, have highlighted the need to use population- and sex-specific data for forensic age estimation [10, 25, 26, 28, 29]. Nevertheless, some of the heterogeneity among studies could be explained by study-specific factors such as the imaging conditions, sample size, age range, and observers’ training and experience [2, 21].

Concerning the probability of an individual being 18 years of age or older (Table 4), our results are consistent with those obtained in similar studies carried out in other populations [22–24, 30–36], with individuals of both sexes exhibiting a higher than 95% likelihood of being at least 18 years of age when the mandibular third molar is fully developed (stage H). Regarding stage G, the probability of being ≥ 18 years was also higher in females than in males in various populations [24, 31, 35]. Therefore, stage H in males and stages G and H in females could be considered good indicators of age of majority in our study population.

For cervical vertebrae, the probability of an individual being older than 18 years was also higher for females than for males when the last stage of development (CVM stage 6) was reached (Table 5). However, these probabilities were lower than those offered by stage H of third molar development for both sexes and by stage G for females. A comparison with similar studies was difficult because they were mostly focused on proposing age estimation equations to provide estimated ages rather than giving probabilities that an individual had reached the age of majority.

The AUC values revealed that radiological examination of the degree of mineralization of mandibular third molars discriminated with high accuracy an age older than 18 years from younger for each third molar assessed (> 0.85, Table 6), in accordance with the values reported in previous studies [25, 37]. According to Swets’ scoring system [38], a diagnostic test can be considered “useful for some purposes” when the AUC value is between 0.7 and 0.9. In our study, tooth 48 exhibited the highest AUC value (0.875) among the studied age indicators and was close to that established by Swets [38] as indicating a “highly accurate” test (> 0.9).

The main aim of forensic tests used to determine the age of majority should be to minimize false positives to avoid mistakenly classifying minors as older than 18 years of age. In this study, as previously found [15, 25, 39], the highest specificity values for third molar maturation were observed for the EFG/H cut-off point, which discriminated between stage H (apices completely closed) and lower maturity scores (Table 6). In our sample, stage H offered the required specificity to avoid ethically unacceptable errors [15], which lead to a violation of minors’ rights, but exhibited significantly decreased sensitivity, which leads to a more benevolent criminal treatment for adults incorrectly classified as minors. Notably, and as reported by others [2], a significant proportion (around 70%) of the individuals in the ≥ 18 age group did not have fully developed third molars (Table 4). In this way, the EF/GH cut-off point reduced the number of false negatives by also considering stage G to be an indicator of age of majority. However, this cut-off point showed a decreased ability to correctly classify minors (i.e., more false positives and lower specificity).

Although somewhat better specificity values were found at certain cut-off points, the combination of cervical vertebrae and third molar development did not improve the accuracy of the method, as reported elsewhere [5] in terms of the period of late third molar development (around 18 years of age). In particular, the large variability in the shapes of the cervical vertebrae that have reached the end stage of development has been identified as a major reason for their lower performance compared with other methods [11]. In our study, the last stage of CVM (stage 6), as well as CVM stages 4 and 5, could be found in both adults (individuals ≥ 18 years of age) and minors (Table 5). In this regard, the overlap in the ranges of the metric characteristics of the cervical vertebrae of adults and children has been indicated as another reason for their limited usefulness for discriminating between ages [11].

## Conclusions

In our study, a fully developed mandibular third molar (Demirjian’s stage H) indicated adult age with 100% certainty. However, a significant proportion of individuals older than 18 years of age had third molars that had not yet completed their development. Thus, immature third molars do not rule out the possibility that an individual has reached the age of majority. The combination of the maturity stages of teeth 38 and 48 or the combination of both with the cervical stages of maturation did not improve upon the prediction accuracy of the mandibular third molars alone. These findings highlight the need to identify other complementary age estimation methods that minimize the number of false negatives obtained with the assessment of third molar development alone. Nonetheless, given our limited sample size, more research is needed to confirm these results.

Future work should also consider potential geographic and sex influences on dental and skeletal development to establish the most appropriate cut-off points for assessing the age of majority in each case. In addition, according to the guidelines of previous studies, an adequate and uniform number of individuals by sex and age group should be included to avoid age mimicry bias.
